# Andrographolide Against Lung Cancer-New Pharmacological Insights Based on High-Throughput Metabolomics Analysis Combined with Network Pharmacology

**DOI:** 10.3389/fphar.2021.596652

**Published:** 2021-04-21

**Authors:** Wen Luo, Li Jia, Jia-Wen Zhang, Dong-Jie Wang, Qiu Ren, Wei Zhang

**Affiliations:** ^1^ Respiratory Department, National Clinical Research Center for Infectious Disease, Shenzhen Third People's Hospital, The Second Affiliated Hospital, School of Medicine, Southern University of Science and Technology, Shenzhen, China; ^2^ Department of Respiratory and Critical Care, First Affiliated Hospital, Harbin Medical University, Harbin, China; ^3^ Department of Respiratory Medicine, Heilongjiang Provincial Hospital, Harbin, China

**Keywords:** target, metabolic pathway, urine biomarker, untargeted metabolomics, lung cancer, liquid chromatography

## Abstract

Andrographolide (Andro) has known to treat various illnesses such as colds, diarrhea, fever and infectious diseases. However, the effect mechanism of Andro is still unclear. Therefore, we used high-throughput metabolomics analysis to discover biomarkers, metabolic profiles and pathways to reveal the pharmacological action and effective mechanism of Andro against lung cancer. The metabolic effects of Andro on lung cancer animal was explored by ultra-performance liquid chromatography-triple-time of flight/mass spectrometry (UPLC-TOF/MS) analysis. Our results showed that Andro exhibited significant protective effects against lung cancer. Compared with control group, a total of 25 metabolites biomarkers was identified in urine of model animals, which 18 of them were regulated toward the normal direction after Andro treatment, and network pharmacology analysis showed that they were related with 570 proteins. Biological pathways analysis showed that the 11 metabolism pathways were regulated by Andro treatment in lung cancer mouse, and amino acid metabolism and arachidonic acid metabolism have great potential as target pathways for Andro against lung cancer. It revealed that high-throughput metabolomics combined with network pharmacology analysis provides deeply insight into the therapeutic mechanisms of natural product for promoting medicine development and disease treatment.

## Introduction

Lung cancer accounting for 20% of all cancer death has been the major murderer for many years, which mostly because it is asymptomatic in primary stage and typically perceived at advanced stages (de Groot et al., 2018; Barta et al., 2019; Kim et al., 2020). The risk factors of lung cancer were related with cigarette smoking, E-cigarettes, biomass fuels, chronic obstructive pulmonary disease, occupational exposures, ambient air pollution, diet and nutrition as well as genetic factors (Trédaniel et al., 1994; Malhotra et al., 2016; Woodard et al., 2016; Sheng et al., 2018). Low-dose chest tomography chest X-rays and sputum cytology screening have been made in clinical practice for early diagnosis, which possesses high rates of positive findings and is appropriate for diagnosis of lung cancers with low threats (Woznitza et al., 2017; New and Keith, 2018). Currently, there are three main treatment methods for lung cancer, which are chemotherapy, radiation therapy and surgery (Wibowo et al., 2016; Azar et al., 2017; Saad and Mathew, 2020). However, chemotherapy brings out adverse effects to a certain extent resulting from a long period management (Gridelli et al., 2011). Radiation therapy is only suitable for patients with small cell lung cancer, and it must be combined with some painkillers during the process of treatment (Forde et al., 2014; Postow et al., 2015; Verma and Simone, 2019). There is an imperative demand to find an emerging method with low side effects and intense activity for lung cancer treatment.

*Andrographis paniculata* (Burm. f.) Nees is a well-known medicinal plant in Southeastern Asian countries, has been widely applied as immunostimulant and anti-inflammatory drugs in clinic practice for many years (Puri et al., 1993). Andrographolide (Andro) is known to possess ability to treat the common cold, myocardial ischemia, respiratory tract infections, diarrhea, inflammation and infectious diseases (Zhu H. L. et al., 2011; Hossain et al., 2014; Wintachai et al., 2015; Ding et al., 2017). Some studies reported that it protected against acute lung injury exerted by reducing expression of myeloperoxidase and neutrophil-derived proteases, increasing in adhesion molecules (Zhu et al., 2013; Yang et al., 2014; Peng et al., 2016a; Gao et al., 2018). It also increases Nrf2 activity to protect against cigarette smoke-induced oxidative lung injury (Guan et al., 2013; Peng et al., 2016b). Andro ameliorates lung inflammation and fibrosis by inhibition of AIM2 inflammasome-mediated pyroptosis, activation of heme oxygenase-1 (Zhu Z. T. et al., 2011; Yang et al., 2013; Gao et al., 2019). The antimicrobial mechanism of Andro is related with up-regulation of human *β*-defensin-2 in human lung epithelial cells (Shao et al., 2012; Tan et al., 2016). It can down-regulate PI3K/Akt signaling pathway in lung cancer cells during in the process of proliferation, migration and invasion (Lee et al., 2010; Lin et al., 2011; Luo et al., 2013; Luo et al., 2014; Lim et al., 2015). Cisplatin-mediated anticancer effects was enhanced by Andro through blockade of autophagy and activation of the Akt/mTOR pathway (Mi et al., 2016; Yuwen et al., 2017).

Metabolomics method can used to discover the biomarker and pathways related to disease processes and elucidate the mechanism of drugs (Johnson et al., 2016; Zhang et al., 2014; Zhang A. et al., 2017; Qiu et al., 2020). The untargeted metabolomics has ability to undertake simultaneous assessment of metabolites without any sample knowledge for hypothesis generation (Liang et al., 2014; Wang et al., 2014; Wu and Feng, 2016; Varma et al., 2018; Zhang. et al., 2019a; Xie et al., 2019). The major disadvantage of untargeted metabolomics is that sample analysis generate lots of data leading to the majority of biological features are unidentifiable (Ribbenstedt et al., 2018; Zhang Y. et al., 2017; Ren, et al., 2018). At present, the combined analytical platform includes the ultra-performance liquid chromatography (UPLC) or gas chromatography in tandem with mass spectrometry (MS) and nuclear magnetic resonance spectroscopy (NMR) (French et al., 2018; Sun et al., 2018; Zhang et al., 2018, Zhang et al., 2019b). These techniques allow for characteristic fingerprints of objects, predictive algorithms with pattern recognition statistical approaches to explain biological effect (Xia et al., 2013; Liang et al., 2015; Zhang et al., 2020). In this work, the untargeted metabolomics strategy based on UPLC-TOF/MS was used to explorethe potential biomarkers and related metabolic pathways and to reveal the anticancer effect of Andro.

## Experimental

### Animals and Feeding

Animal care and experimental procedures were performed in accordance with the criteria outlined in the “Guide for the Care and Use of Laboratory Animals” prepared by the National Academy of Sciences. A total of forty-seven male C57BL/6 mice (6–8 weeks old, 20 ± 2 g weight) in SPF grade were purchased from Envigo Laboratory Animal Co., Ltd (Suzhou, China, catalog no. SCXK 2019-0002), which were bred and maintained in pathogen-free cages with 12 h light/dark cycles from 9:00–21:00, temperatures of 24°C ± 2°C, humidity of 50 ± 5%, and food and water ad libitum.

### Reagents

Pentobarbital sodium, sodium chloride solution and neutral buffered formalin were purchased from Xinxiang Sanwei Disinfectant Co., Ltd. (Tianjin, China). Andro (97.5% purity) was provided by Northern Biotechnology Research Institute (Beijing, China) and its chromatographic fingerprint of HPLC is shown in Supplementary Figure S1. Cisplatin was purchased from APIChem Technology (Hangzhou, China) and used as the positive drug. Interleukin-6 (IL-6), interleukin-2 (IL-2) and interleukin-10 (IL-10) were was purchased from Toronto Research Chemicals (Toronto, Canada). Interleukin-1 beta (IL-1β) was obtained from Origene Technologies, Inc (Beijing, China). Tumor necrosis factor-α (TNF-α) and nuclear transcription factor-κB (NF-κB) were bought from Biotechnology BioengineeringCo., Ltd. (Shanghai, China). Immunoglobulin G (IgG), immunoglobulin A (IgA) and immunoglobulin M (IgM) were purchased from Bioworld Technology, Inc (St Louis Park, MN, United States). Vascular endothelial growth factor (VEGF) was purchased from Jackson ImmunoResearch (West Grove, PA, United States). Hypoxia-inducible transcription factor-1α (HIF-1α) and matrixmetallo proteinase-2 (MMP-2) were purchased from BIOSS (Beijing, China). Interferon-γ (TFN-γ), transforming growth factor-β (TGF-β), toll like receptor 4 (TLR4) and myeloid differentiation factor 88 (MYD88) were provided from BioswarmBiotechnology Co., Ltd. (Hangzhou, China). Methanol, acetonitrile and formic acid were of chromatographic grade and purchased from Fisher Chemical Company (Geel, Belgium). Pure water was brought from the A.S. Watson Group Ltd. (Hong Kong, China). Chromatographic grade leucine enkephalin were purchased from Invitrogen Life Technologies (Carlsbad, CA, United States).

### Instrument

High-performance ultra-performance liquid chromatography-Triple-time of flight/mass spectrometry (UPLC-Triple-TOF/MS) system used in this research consisted of a ACQUITY H-CLASS UPLC (Waters Corp., Milford, MA, USA) and a Triple TOF™ 5,600 + Mass Spectrometer detector equipped with positive and negative modes of electrospray source (AB SCIEX, Foster City, CA, United States). H3018DR cryogenic high-speed centrifuge (Sigma Laborzentrifugen GmbH, Osterode am Harz, Germany); Normal-temperature centrifuge (Scientific Industries Inc., Bohemia, NY, United States)); WH-861 Vortex Shaker (Tanon Science andTechnology Co. Ltd. China); CS-6400 automatic biochemical analyzer (Vital Scientific, Eppendorf, Germany)); BCD-206TAS Low-temperature refrigerator (Haier Company, China); AE240 mettler electronic balance (Mettler Toledo, Columbus, Ohio, United States).

### Grouping, Modelestablishment and Administration

After seven days of adaptive feeding, C57BL/6 mice were divided into four groups in the light of the principle of weight uniformity: normal control group (NC, n = 10), lung cancer model group (LC, n = 13), cisplatin-treated group (LC + Cis, n = 12) and Andro-treated group (LC + Andro, n = 12). Lewis lung carcinoma cells were obtained from the Cancer Center of West China Medical University (Sichuan, China), then were cultured and generated in Dulbecco's modified Eagle’s medium (Thermo Fisher Scientific, Inc., Waltham, MA, United States) containing 10% fetal bovine serum (Thermo Fisher Scientific, Inc.). Under 37°C and 5% CO_2_ saturated humidity, the medium was changed every other day. Cells were every digested by trypsin, and the logarithmic phase cells were collected for experiment. A single dose 1 × 10^7^/ml Lewis lung cancer cells 0.2 ml were inoculated subcutaneously into the light axillary of C57BL mice. Tumors with a diameter of approximately 1–1.5 cm were formed in the right armpit of the modeled mice (10 days following injection), which the mice were considered as successfully established in xenografts manner (Li et al., 2016; Zhang Y. et al., 2019; Zhao et al., 2019). From the first day of modeling, mouse in NC and LC group were received dosage of 0.2 ml/10 g physiological saline via intragastric administration, mouse in LC + Cis group and LC + Andro group were respectively received dosage of 4.0 mg/kg/day cisplatin and 10.0 μMol/molar/kg/day Andro in intragastric administration way for twenty-eight days.

### Sample Collection and Preparation

#### Urine Sample

After the final time of Andro administration, each mice in NC, LC, LC + Cis and LC + Andro group was individually fed in metabolic cages to gather urine for 24 h. The urine samples were centrifuged at 10,000 g for 15 min at 4°C, and then the supernatant liquid were delivered into a new eppendorf tube stored in −80°C refrigerator until metabolomics study. Prior to analysis, urine samples were thawed at 4°C until no ice was visible in the sample. Subsequently, 100 μL of aliquots of the urine samples were added 400 μL methanol in order to precipitate the proteins. The solution mixture was vibrated for 60 s and centrifuged at 12,000 g for 15 min, which gained supernatant was evaporated to dryness at 60°C under a stream of nitrogen. The residue was dissolved again in 150 μL of methanol followed by vibrated for 30 s and centrifuging at 12,000 rpm for 10 min. 10 μL clear supernatant from each mice were mixed into a new eppendorf tube as quality control (QC) sample and the remained samples were passed through a 0.22 μm PTFE filter for UPLC-Triple-TOF/MS analysis.

#### Serum Sample

At 24 h after the final time of Andro treatment, mouse in NC, LC, LC + Cis and LC + Andro group were mildly anesthetized with sodium pentobarbital (50 mg/kg, i.p.). The blood samples of the animals were respectively collected from the abdominal aorta by a syringe, which were placed 10 min for coagulation and centrifuged at 3500 g for 15 min at 4°C. The obtained supernatant serum was delivered into a clean plastic tube and stored under −80°C until blood biochemical analysis.

#### Tumors Tissue Sample

Mice in each group were sacrificed by cervical spine removal, and the axillary subcutaneous tumor tissue was quickly and completely peeled off on ice. After weighting, the tumor samples were placed in liquid nitrogen and stored in a refrigerator at −80°C for tissue biochemical analysis.

### Biochemical Indexes Detection

Prior to analysis, serum and tumor samples were thawed at 4°C until no ice was visible in the sample. According to the manufacturer’s instructions, serum biochemical parameters level of IL-6, IL-1β, TNF-α, VEGF, HIF-1α, MMP-2, IgG, IgA, IgM, IL-2 and TFN-γ were evaluated using ELISA kits. Tumor tissue samples were homogenized and dissolved by corresponding solution based on the manufacturer’s instructions of kits, then the IL-10, TGF-β, TLR4, MYD88 and NF-κB level were measured by automatic biochemical analyzer.

### Metabolomics Analyses

#### Chromatography and Mass Spectrometry Conditions

All urine samples were analyzed using the UPLC-Triple-TOF/MS system following the manufacturer’s instructions. An Acquity UPLC HSS C18 column (1.7 μm, 2.1 × 100 mm^2^) from Waters Corporation (Massachusetts, United States) was used for chromatographic separation. The column oven was kept at 33°C, and the temperature of the sample manager was maintained at 15°C. The flow rate was set 0.3 ml/min and the injection volume was 3 μL. 10 and 90% acetonitrile aqueous solutions were applied as weak and strong wash solvents respectively in the analyzed process. The mobile phase consisted of A (0.1% formic acid−water) and B (0.1% formic acid−acetonitrile). UPLC elution conditions were run as following: 0–1.5 min, 8% B; 1.5–4 min, 8–35% B; 4–8 min, 35–70% B; 8–9 min, 70–90% B; 9–11 min 90–8% B; 11–13 min 8% B. QC samples were sampled six times before analysis, and then was injected once every eight experimental samples. Using the Triple-TOF/MS model, the electrospray ionization (ESI) source was operated in both positive and negative modes. 50 to 1200 Da mass spectrum data were collected in MSE centroid mode. Accurate mass determination using leucine-enkephalin (m/z 556.2771 in ESI^+^and 554.2615 in ESI^−^) was considered as external reference for Lock Spray™ injected at a flow of 10 μL/min in order to ensure the mass reproducibility and accuracy. The optimized MS parameters in the positive-ion detection mode are as follows: source temperature, 140°C; desolvation temperature, 460°C; desolvation gas flow 800 L/h; capillary voltage, 2.6 kV; cone voltage, 45 V, cone gas flow, 55 L/h, collision energy, 15–55 eV. The negative ion mode parameters was the same as the positive-ion detection mode, except for being negative in the capillary voltage 2.0 kV and cone gas flow 45 L/h. In addition, the air curtain gas was set 40 psi; 55 psi of atomizing gas and 55 psi of auxiliary atomizing gas.

#### Metabolome Data Interpretation

MarkerLynx XS Version 4.1 software (Waters Co., Milford, MA, United States) was used to command the instrument system, perform the sample list and obtain raw data in m/zXML format, and then XCMS (www.bioconductor.org/) was applied to extract the peak data, peak matching, peak alignment, and export before multivariate statistical analysis of variables. Three-dimensional data matrix including sample identity (ID), molecular mass (MS), peak area, and standardized ion strength was saved after data preprocessing by unit variance scaling and the mean-centered method. The exported data were imported to SIMCA-P software (Version 14.1, Umetric, Umea, Sweden) for multivariate analysis such as principal component analysis (PCA) and orthogonal projections to latent structures discriminant analysis (OPLS-DA). PCA is an unsupervized method of pattern recognition approach that have ability to gain the overview and classification showing maximum variation and pattern recognition between. A score plot of the OPLS-DA model as supervised method was employed to visualize the metabolic difference between two different groups. S-plots generated from the OPLS-DA predictive results probe into the potential biomarkers that made a remarkable contribution to the metabolic distinction, which ion with the variable importance in the projection (VIP) value above 1.0 were considered significant. Meanwhile, data between two different groups were dissected by two-way analysis of variance to test the significance of differences, which significant differences meet p values less than 0.05 in test were considered significant. Afterward, the potential ions were verified by the raw MS data in chromatogram and accurate masses of quasimolecular ions were exported into biochemical databases online including METLIN (http://metlin.scripps.edu/), SMPD (http://www.smpdb.ca/), HMDB (http://www.hmdb.ca/) and KEGG (http://www.kegg.com/) (5 ppm as the accepted mass error) to confirm the structure of biomarkers. Next, the biomarkers were further identified by comparing the retention time and the tandem mass spectrometry (MS/MS) fragments of metabolites with those of the chemical standards. Adducts were obtained with the mass tolerance at 10 ppm. MetaboAnalyst 5.0 (http://www.metaboanalyst.ca/) was used to seek out vital potential metabolic pathways enrichment and topological analysis and network establishment. The metabolic correlation protein analysis of Andro efficacy was performed by Cytoscape 3.7.1 and Gene Cards (https://www.genecards.org/).

### Statistical Analysis

During the experiments, all the tests were carried out at least three times using independent samples. All data are presented as mean ± standard deviation, which statistical analysis was conducted using SPSS software, version 12.0 software (SPSS, Inc., Chicago, IL, United States). The Student's t-test was applied to compare the difference between two individual groups, then P-values ≤0.05 were considered to indicate a statistically significant and P-values ≤ 0.01 were considered to indicate statistical significant. All statistical analyses were conducted on GraphPad Prism 6.05 software.

## Results

### Effect ofAndro on Biochemical Index

The mouse in NC group with smooth and shiny hair present normal feeding, drinking, excretion and weight gain in active state. In LC group, mouse with dirty and messy fur have a decreasing drinking, feeding and increasing excretion, moist cage, and weight reduction. Compared with LC group, the general states of the mouse in LC + Andro and LC + Cis is being made better.

**FIGURE 1:**
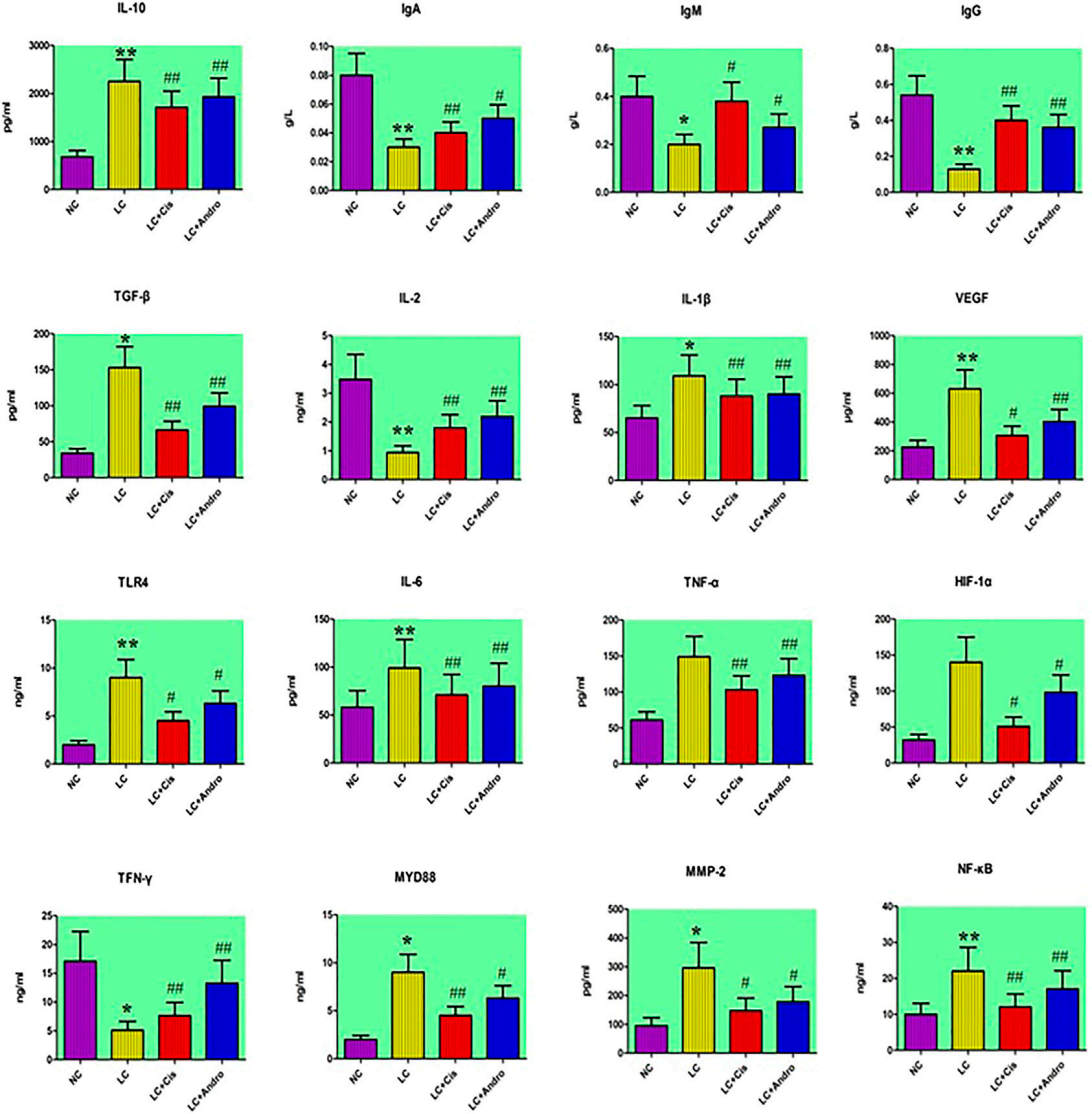
The changes of chemical indexes content in different groups after Andro administration. “*” LC group vs NC group, *p* < 0.05; “**” LC group vs NC group, *p* < 0.01; “#” LC + Cis or LC + Andro group vs LC group, *p* < 0.05; “##” LC + Cis or LC + Andro group vs LC group, *p* < 0.01.

As shown in Figure 1, compared with the NC group, the serum content of IL-6, IL-1β, TNF-α, VEGF, HIF-1α and MMP-2 from the LC group were increased, and IgG, IgA, IgM, IL-2 and TFN-γ level were decreased. Meanwhile, IL-10, TGF-β, TLR4, MYD88 and NF-κB in tumor tissue were increased, indicating that the LC model of mouse was successfully developed. After therapeutic period, Andro could remarkably reduce IL-6, IL-1β, TNF-α, VEGF, MMP-2 level in blood and IL-10, TGF-β, NF-κB level of tumor tissue with significantly statistical implications (*p* < 0.01). The content of serum MMP-2, tumor tissue TLR4, MYD88 were also reduced by Andro treatment with statistical implications (*p* < 0.05). In addition, the level of IgG, IgA, IgM, IL-2 and TFN-γ in LC + Andro groups was significantly higher than those in the model group. Among them, the blood IgG, IL-2 and TFN-γ possess significantly statistical implications (*p* < 0.01) compared with LC group. Mouse in LC + Cis and LC + Andro group showed similar trends in biochemical indicators, indicating that Andro has a certain therapeutic effect on lung cancer, mainly by blocking the body's inflammatory response, promoting the regulation of the immune system, inhibiting the generation of cardiovascular disease, and preventing the proliferation, differentiation and metastasis of cancer cells.

### Metabolic Profiling Analysis

**FIGURE 2:**
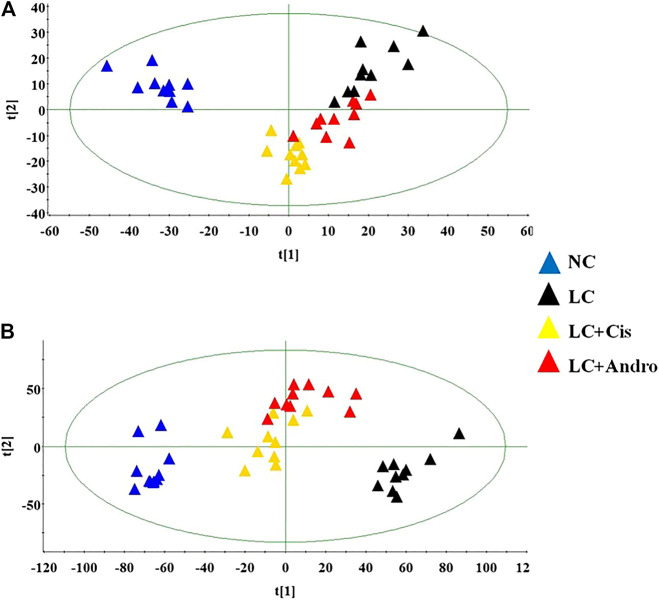
The score plot of the PCA model among NC, LC, LC + Cis and LC + Andro group in positive ion mode **(A)**; The score plot of the PCA model among NC, LC, LC + Cis and LC + Andro group in negative mode **(B)**.

Under the abovementioned condition, urinary sample from different groups could present in excellent peak shape, temperate intensity and clearly separation. In the initial stage, nine chromatographic peak were selected in overlaid chromatograms of the QC, which the relative standard deviation (RSD) of the peak area and retention time are respectively less than 5% indicating that the detection method possesses well repeatability. Due to spectra complication, the discrimination between each group were not clearly highlighted. Multivariate statistical analysis was applied to discern the discrepancy of metabolic components among the four groups. In Figures 2A,B, the positive and negative mode plots of urinary samples are shown that four group exhibit the obvious separation and did not exceed the limit indicating metabolic differences among the different four groups is significant and anomalous sample was not existed in the clustering of data. The samples from the NC group clustered together and remained relatively far from those from the LC groups. In addition, the clustering of LC + Cis group and LC + Andro group remained relatively far from LC group and close to NC group. Compared with LC + Andro group, the clustering of LC + Cis group is more close to NC group. The results suggested that metabolic state of LC mouse could be regulated by Andro treatment and the further multivariate analysis was necessary to explore potential relationships.

### Biomarkers Screening, Discovery and Identification

**FIGURE 3:**
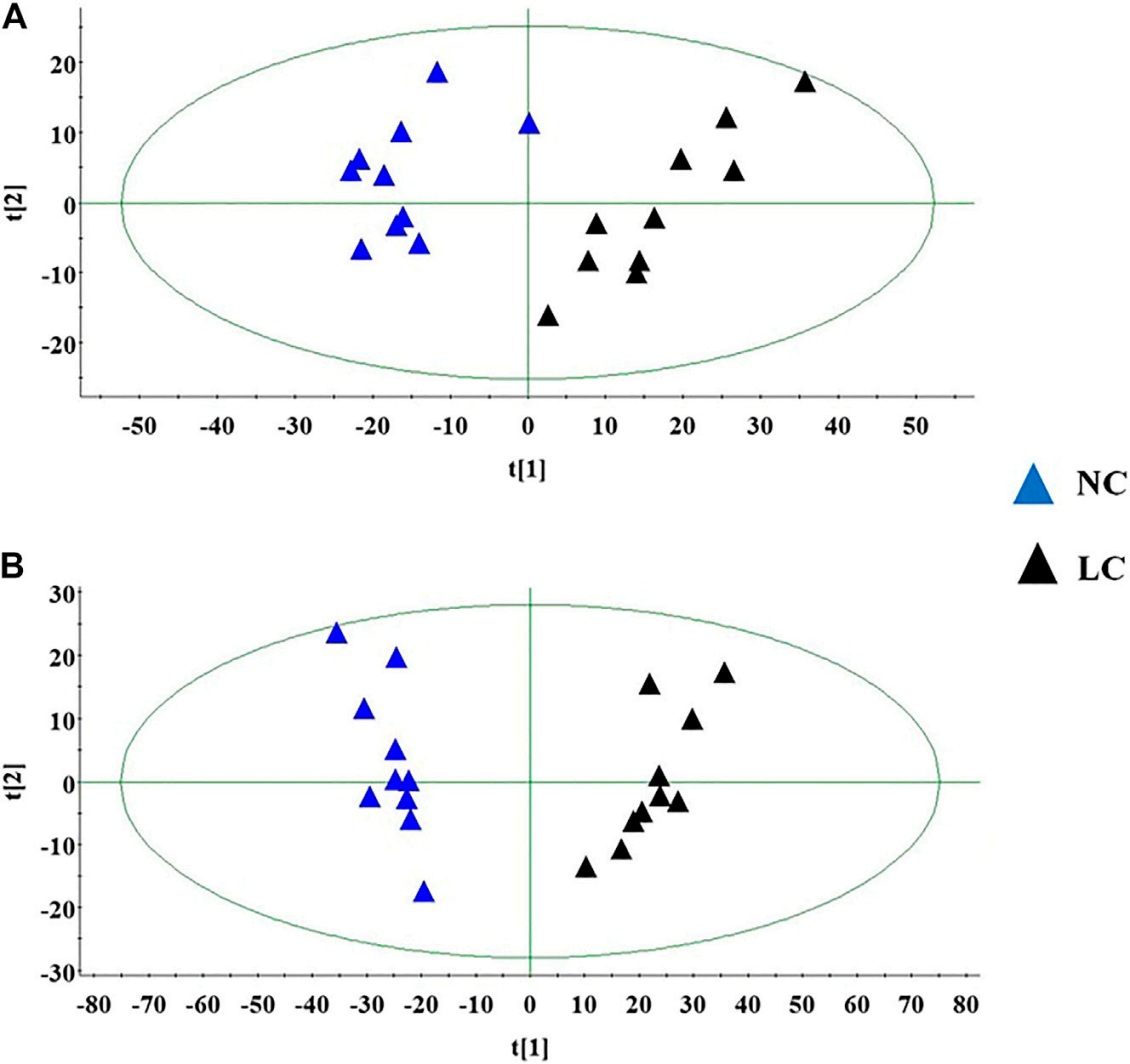
The score plot of the PCA model between NC and LC group in positive ion mode **(A)**; The score plot of the PCA model between NC and LC group in negative mode **(B)**.

**FIGURE 4:**
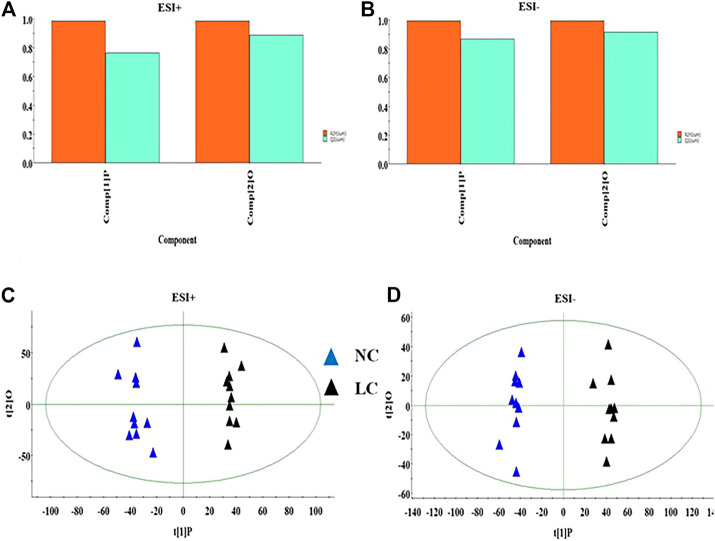
The goodness of fit test for OPLS model in ESI+ and ESI- mode **(A,B)**. OPLS-DA analysis of the data derived from the ESI+ and ESI- mode between NC and LC group **(C,D)**.

**FIGURE 5:**
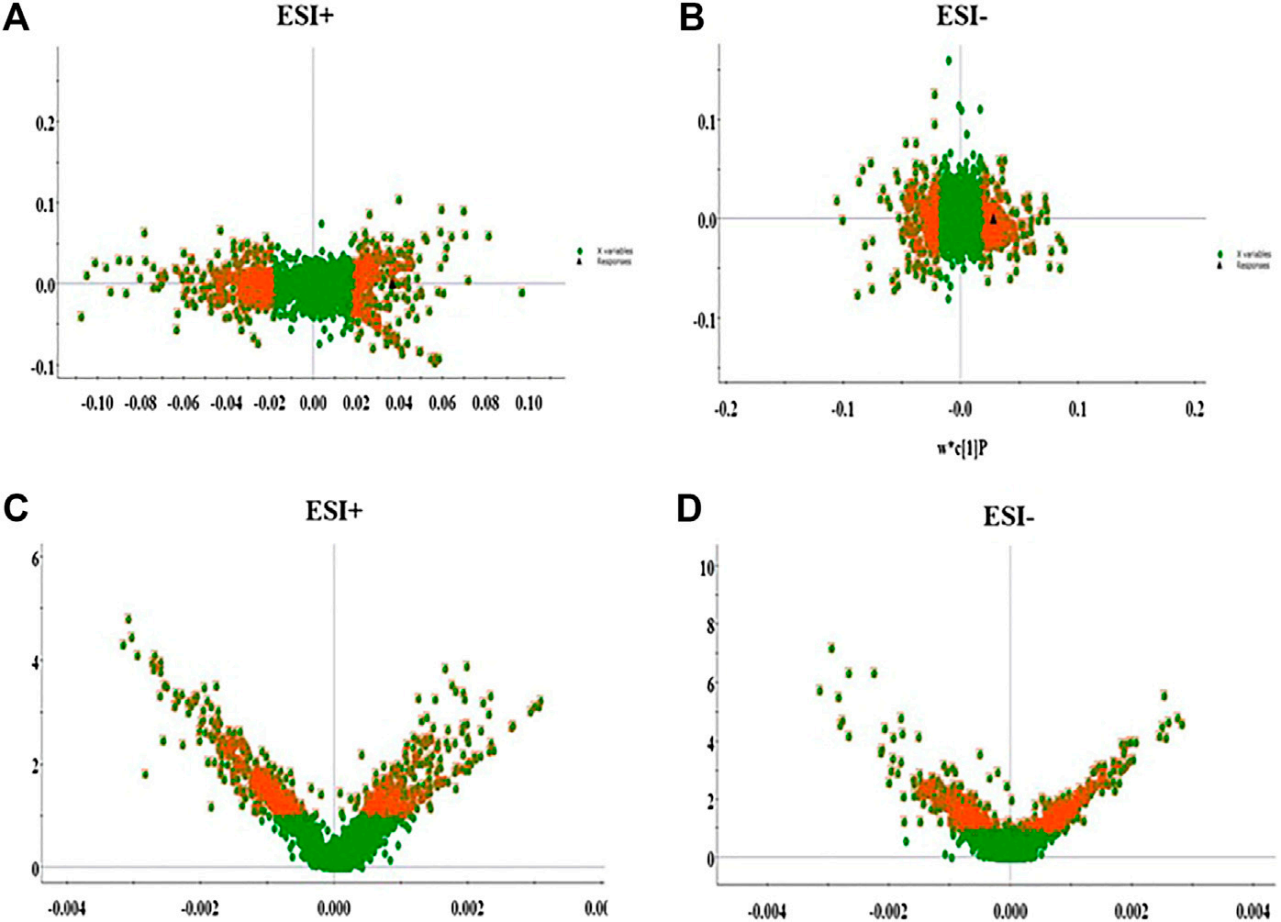
The loading plot for OPLS model between NC and LC group in ESI+ and ESI- mode **(A,B)**. VIP plot for OPLS model between NC and LC group in ESI+ and ESI- mode **(C,D)**.

Firstly, the data of NC and LC group were separately compared in both ion mode as shown in Figures 3A,B, which there are evident separation between the clustering of NC group and LC groups, and the dispersion within the group is relatively clear. Secondly, a cross validation test that was performed the calculation of the R2 and Q2 values to evaluate the goodness of fit of the OPLS models, which R2 close to 1 is the requisite condition for a good model, and Q2 more than 0.5 represent good predictability of the model. In Figures 4A,B goodness of fit test was carried out to assess the predictability of the model indicating that the model have a well goodness. The clustering of LC group can be obviously separated from NC group (R2Y (cum) = 0.970 and Q2 (cum) = 0.791 in the ESI + model, and R2Y (cum) = 0.982 and Q2 (cum) = 0.738 in the ESI- model) as shown Figures 4B,C. The mass-to-charge ratio with large dispersion in the statistical analysis of the loading plot acts a vital role in the separation between groups, the loading plot generated from OPLS-DA model as shown in Figures 5A,B bring out the ions with major differences in abundance between NC group and LC group. From the Figures 5C,D of volcano plot, the VIP value was larger, the contribution was greater. Potential metabolite marker selection need to simultaneously satisfy the strength of both contribution and variable reliability from the same OPLS-DA model, which the value of the VIP score is more than 1.0 and p value is less than 0.05 in Student’s t-test.

Combined with the retention time, exact mass-to-charge ratio of the variables and online databases, 25 urine meatbolites including valine, inositol phosphate, alanine, thymine, proline, L-glutamine, pyridoxic acid, 3-hydroxybutyric acid, arginine, arachidonic acid, xanthurenic acid, glucose, isoleucine, p-cresol sulfate, kynurenic acid, tyrosine, chenodeoxycholic acid, creatinine, phenylpyruvic acid, coproporphyrin III, 12,13-EpOME, glycyl-threonine, 9 (S)-HPODE, 3-oxohexadecanoic acid, lactic acid were identified as biomarkers associated with the metabolic disturbances in animals with the lung cancer. The basic information such as molecular formula, compound name, corresponding m/z, VIP value was listed in Supplementary Table S1. Among them, specific content changes of 18 metabolites were determined to be changed trend back to NC group level after treatment with Andro, which eight metabolites including 12,13-EpOME, creatinine, inositol phosphate, lactic acid, thymine, arginine, coproporphyrin III and arachidonic acid were down-regulated in the urine, and ten metabolites including alanine, L-glutamine, isoleucine, 3-hydroxybutyric acid, proline, valine, tyrosine, xanthurenic acid, kynurenic acid and p-cresol sulfate were up-regulated as shown in heatmap of Supplementary Figure S2A. Detailed the comparisons of metabolite relative peak area in NC, LC, LC + Andro and LC + C is group are shown in Supplementary Figure S2B, which Andro treatment has a greater influence on the content of isoleucine, 3-hydroxybutyric acid, arginine, coproporphyrin III, alanine, L-glutamine, lactic acid, arachidonic acid with significantly statistical implications (*p* < 0.01).

### Metabolic Pathways Regulated by Andro

**FIGURE 6:**
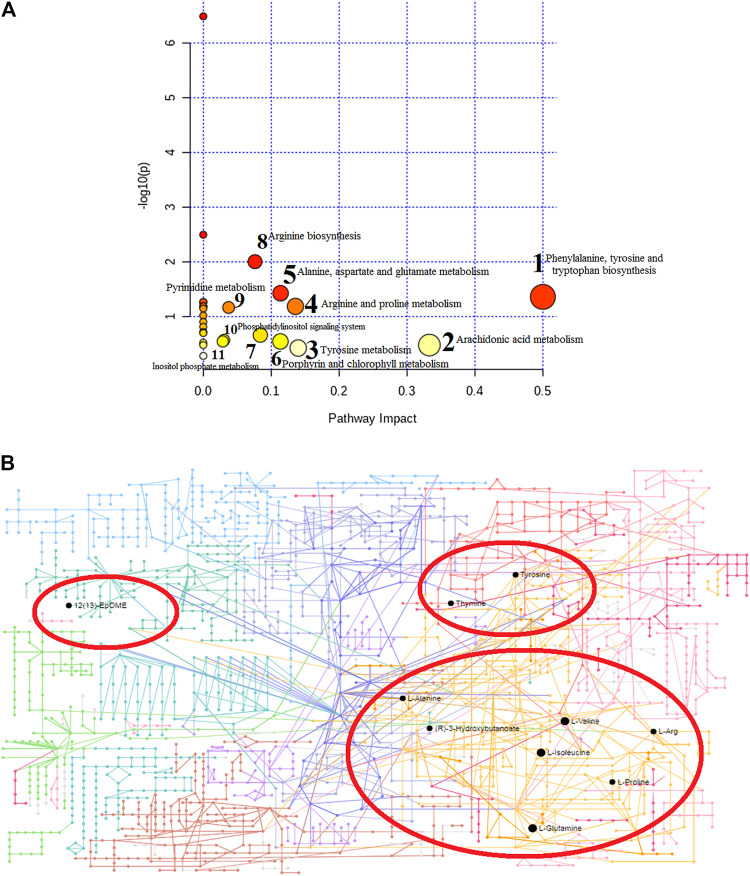
Metabolic pathway analysis of identified 18 differential metabolites after Andro treatment, and impact value more than zero of pathways name **(A)**; KEGG global metabolic network potential metabolites in model mouse after Andro administration **(B)**.

The changes in the levels of potential biomarkers suggested that the metabolic disturbances in mouse with lung cancer were relieved by Andro referring to phenylalanine, tyrosine and tryptophan biosynthesis, arachidonic acid metabolism, tyrosine metabolism, arginine and proline metabolism, alanine, aspartate and glutamate metabolism, porphyrin and chlorophyll metabolism, pyruvate metabolism, arginine biosynthesis, pyrimidine metabolism, phosphatidylinositol signaling system and inositol phosphate metabolism after Pareto method to standardize the data. As shown in Figure 6A, the impacts on the pathways phenylalanine, tyrosine and tryptophan biosynthesis and arachidonic acid metabolism were stronger, where the metabolic pathway with the influence value greater than or equal to 0.30 can be selected as the potential key metabolic pathway of drugs. From KEGG global metabolic network of Figure 6B, potential metabolites regulated after Andro administration were closed with valine, leucine and isoleucine biosynthesis, alanine, aspartate and glutamate metabolism, phenylalanine, tyrosine and tryptophan biosynthesis, etc.

### The Target Prediction

**FIGURE 7:**
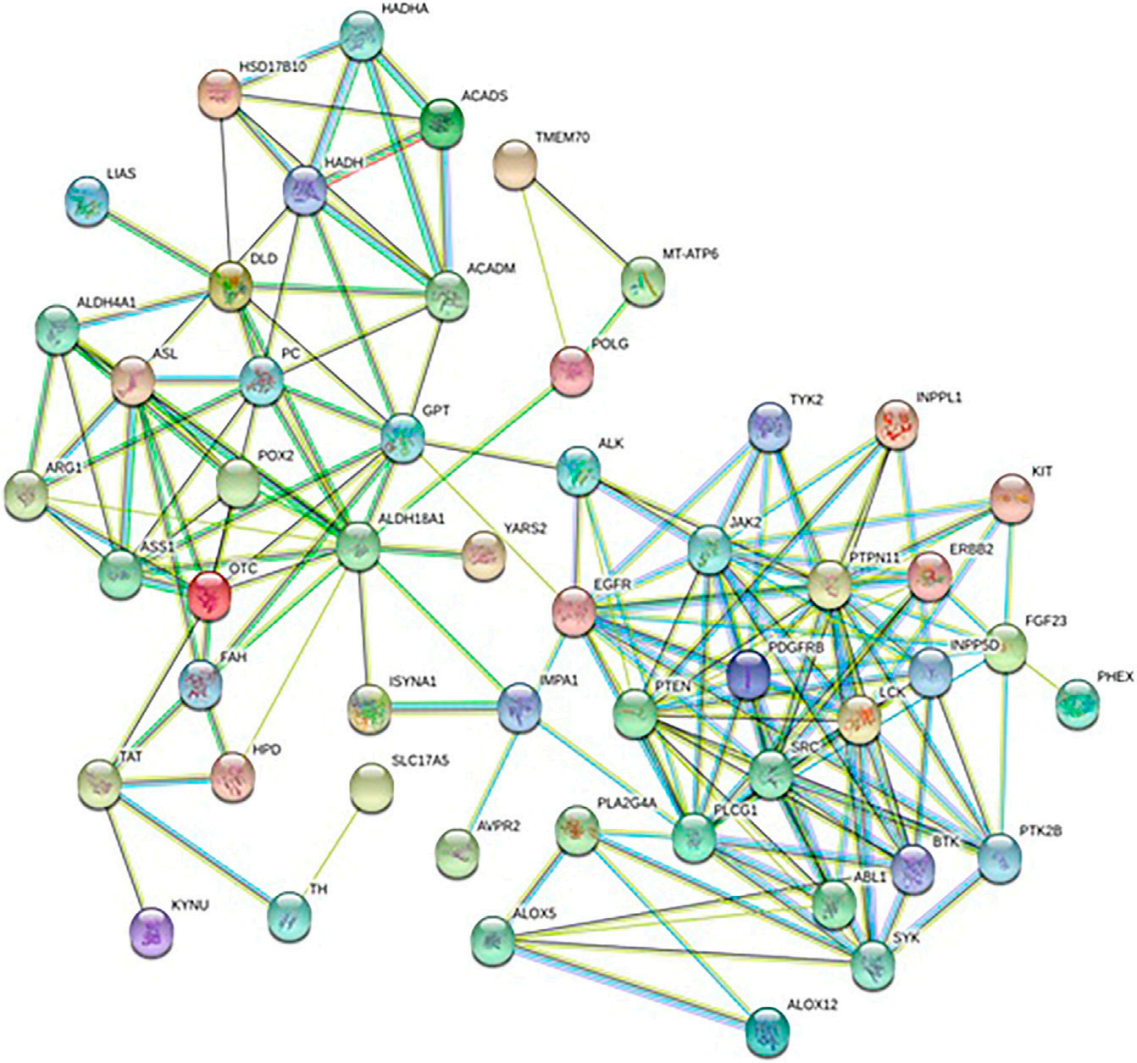
Protein-protein correlation analysis of differentiated metabolites involved in Andro treatment protecting against lung cancer mouse.

A total of 570 proteins were predicted and closely related with metabolites regulated by Andro, which mainly involves amino acid metabolism and arachidonic acid metabolism. ASS1, TAT, ALDH4A1, PTPN11 and JAK2in Figure 7 has higher correlation degree considered as potentially important markers for further study.

## Discussion

In this study, the biochemical analysis and pathological studies have shown that Andro treatment can enhance the immune system function of lung cancer model animals, inhibit inflammation reaction, tumor cell growth and metastasis. TNF-α is an inflammatory factor with multiple types of biological effects, which is secreted by activated macrophages, monocytes and T cells to mediate the process of inflammation and directly participate in the process of lung tissue injury and apoptosis (Inui et al., 2018). IL-6 secreted by monocytes and macrophages is an inflammatory mediator involved in the immune regulation of infection and tumors (Zhao et al., 2018). IgM with a large molecular weight cannot pass through blood vessel walls, and can be used for early diagnosis of body infections (Macpherson et al., 2008; Liu and May 2016; Hansen et al., 2019; Zhou et al., 2019). TLR4 is a member of the Toll-like receptor superfamily that plays a biological role in the form of binding to ligands (Zhang J. et al., 2019). Tumor cells can release a large number of cytokines in the process of immune remodeling, such as IL-10 and TGF-β, the latter can weaken the killing activity of cytotoxic T lymphocytes and natural killer cells, evading immune surveillance and promote tumor metastasis (Solinas et al., 2010; Wei et al., 2010; Bellomo et al., 2016; Anguiano-Hernandez et al., 2019; Dong et al., 2019).

**FIGURE 8:**
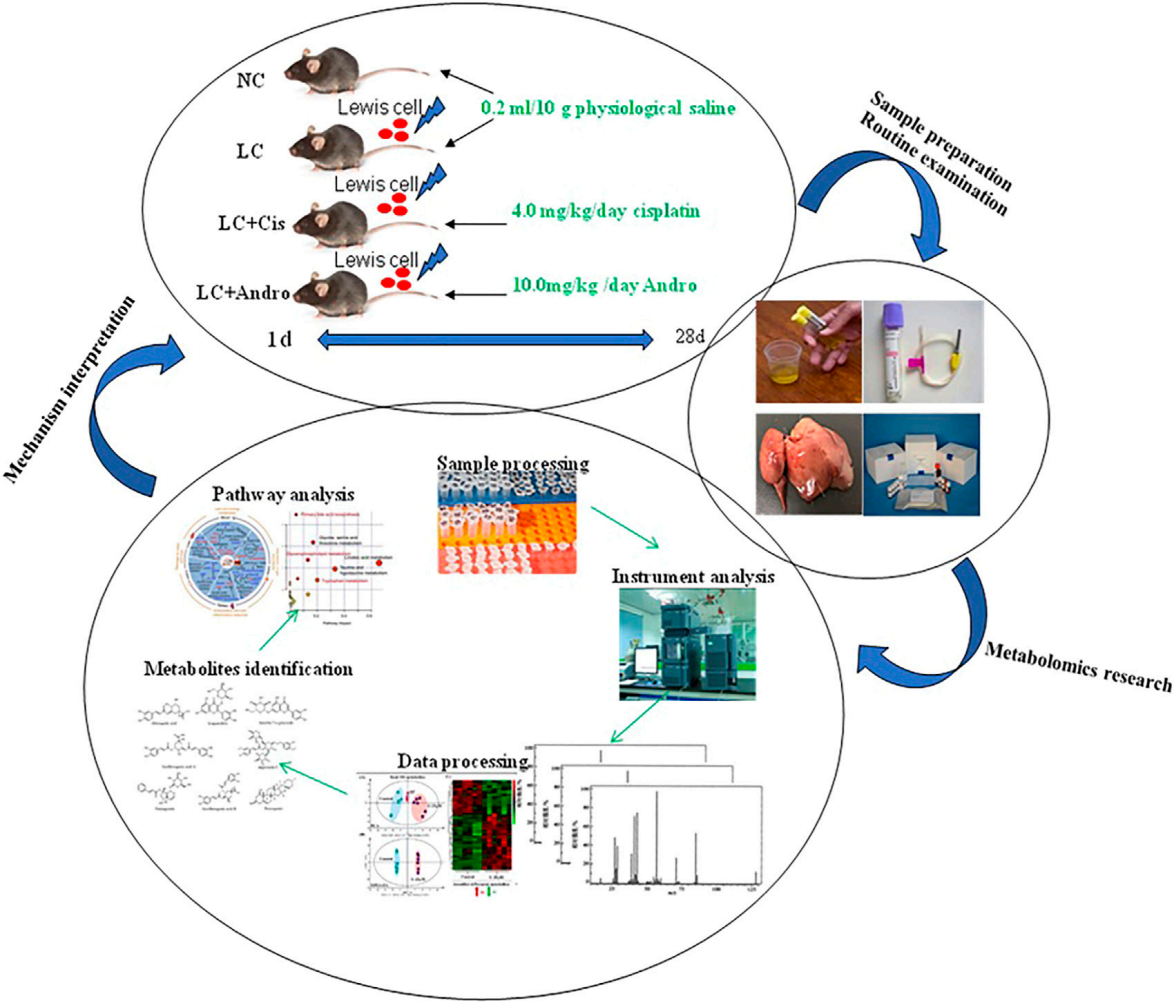
A flow chart for the experiments.

A flow chart for the experiments was shown in Figure 8. According to urine metabolomics analysis, Andro can regulate 18 of 25 biomarkers associated with the pathogenesis of lung cancer, including alanine, L-glutamine, isoleucine, 3-hydroxybutyric acid, 12,13-EpOME, arginine, proline, valine, tyrosine, creatinine, inositol phosphate, lactic acid, thymine, xanthurenic acid, kynurenic acid, p-cresol sulfate, coproporphyrin III, arachidonic acid, which is mainly related to phenylalanine, tyrosine and tryptophan biosynthesis, arachidonic acid metabolism, arachidonic acid metabolism, arginine and proline metabolism, alanine, aspartate and glutamate metabolism, porphyrin and chlorophyll metabolism, pyruvate metabolism, arginine biosynthesis, pyrimidine metabolism, phosphatidylinositol signaling system and inositol phosphate metabolism. In the light of the impact value of the metabolic pathway, the pharmacological action and effective mechanism of Andro mainly acts on the amino acid metabolism pathway and arachidonic acid metabolic pathway to protect against lung cancer.

Amino acid metabolism not only plays an important role in the body's anabolism, but also plays an important role in the proliferation, apoptosis and invasion of tumor cells. Some amino acids are considered as tumor markers and present abnormal expression in patients with different tumors of lung cancer, skin cancer, prostate cancer, colon and breast cancer (Zhang et al., 2013; Liang et al., 2016; Nan et al., 2016; Lukey et al., 2017). However, the abnormal state is not much different from normal people in chronic wasting diseases. During the process of tumors growth in the lung, it not only affects the human respiratory and circulatory system, but also causes changes in the body’s energy metabolism even the overall metabolic state leading to some amino acid metabolism-related enzymes alteration in the body. The abnormal expression of amino acids metabolism provides energy for tumor tissues, constructs related proteins required for their growth and development, then it can also escape the tumor cell killing effect and immune surveillance of the host immune system (Vettore et al., 2020). Amino acids can also be used as signal molecules to participate in various signal pathways of tumor cells, which regulate themselves to take part in the formation of cellular energy-related metabolic regulation signal pathways, and control cell proliferation, growth and Invasion ability as downstream proteins of proto-oncogenes (Liang et al., 2017; Sivanand and Vavder Heiden, 2020). Alanine is a non-essential amino acid produced by glycogen in the human body, which is produced by the conversion of carbohydrate pyruvate or the decomposition of DNA, dipeptide carnosine and goose serotonin. It can be converted into pyruvate and tricarboxylic acid cycle intermediates, and then converted into glucose through gluconeogenesis, as an energy source to meet the huge energy demand consumed by various metabolic activities of tumor cells. When branched chain amino acid is insufficient, alanine level is usually decreased, which may be related to muscle metabolism. Alanine can promote energy synthesis in cells and provide sufficient energy for cell growth (Deberardinis et al., 2008). It is an inhibitory neurotransmitter in the brain as the same as GABA, taurine and glycine. In addition, tumor cells also use glutamine as another source of energy. As the main substrate of aerobic metabolism of tumor cells in mitochondria, the proliferating tumor cells need to consume a large amount of glutamine (Bathe et al., 2011). Some clinical trials have demonstrated that patients receiving glutamine supplementation have higher nitrogen balance, and polymorphonuclear neutrophil granulocytes producing cysteyl-leukotrienes, lymphocyte recovery and intestinal permeability have been improved. Glutamine is converted to glutamate by the reaction of glutaminase and amidase. The glutamate product can be directly incorporated into GSH, or enter the Krebs cycle as 2-oxoglutarate through transamination or oxidative deamination reactions. Subsequently, the OAA formed in the Krebs cycle is transamination to aspartic acid, which is removed from the Krebs cycle for pyrimidine biosynthesis (Deberardinis et al., 2008; Bathe et al., 2011). This study revealed that the levels of alanine and glutamine in the model group were reduced, and Andro could restore the level of alanine and glutamine content to the control group trend by regulating alanine, aspartate and glutamate metabolism, and pyrimidine metabolism. Arginine, as an essential amino acid, is synthesized in the urea cycle of adults. It helps to process ammonia and can be converted into glucose and glycogen when needed. Arginine can activate AMP kinase, and then stimulate skeletal muscle fatty acid oxidation and muscle glucose uptake leading to the increasing level of the insulin secreted by pancreatic β cells. It also is involved in the metabolism of nitric oxide that is a vasodilator and free radical used by nitro-oxidative stress, apoptosis, cell cycle, angiogenesis, invasion and metastasis. Therefore, arginine deprivation may provide a potential treatment for lung cancer (Yang et al., 2009; Grimm et al., 2013). In our study, it was found that the arginine content in the urine of lung cancer mice reduced after Andro treatment, which was related to the regulation of arginine and proline metabolism as well as arginine biosynthesis. Proline is a non-essential amino acid synthesized from glutamic acid. It is an important part of collagen and has potential endogenous excitotoxin/neurotoxin activity, which can act as a neurotoxin and metabolic toxin to damage nerve cells and nerve tissue causing adverse health effects when it keeps at a high level for a long time. According to reports, the plasma concentration of proline in the model group was significantly reduced compared with the control group, and the rapid increase of proline dehydrogenase transcription by the tumor suppressor p53 triggered the degradation of this amino acid in cancer (Zhao et al., 2014; Phang et al., 2015). After Andro treatment, the proline content in the urine of lung cancer mice was increased medicated by regulating arginine and proline metabolism. Tyrosine, like other amino acids, is a component of protein and an alternative energy source for cell function. The liver is the main organ where tyrosine degradation occurs, producing intermediates or precursors for gluconeogenesis and ketone production. The degradation of tyrosine is catalyzed by a series of enzymatic reactions, which tyrosine metabolism disorders are related to many diseases, such as Huntington's disease and esophageal cancer. In the catalytic reaction of phenylalanine hydroxylase, tyrosine can be metabolized to phenylalanine. The lack of polycyclic aromatic hydrocarbons or the decrease of liver activity can cause phenylalanine metabolism disorder and acute liver damage. The decreasing level of tyrosine in urine of the model group can infer that the liver function in the lung cancer body is abnormal (Wiggins et al., 2015; Herman et al., 2019). Andro can adjust phenylalanine, tyrosine and tryptophan biosynthesis, and tyrosine metabolism to promote tyrosine level close to normal state.

Tumor cell membrane phospholipids can release arachidonic acid (AA) through the action of phospholipase A2 (PLA2), then it was catalyzed to produce eicosanoids such as prostaglandins (PGE), leukotrienes and hydroxyeicosatetraenoic acid (HETEs) through the key enzymes from arachidonic acid metabolism network such as cyclooxygenase (COX), lipoxygenase (LOX) and cytochrome P450 monooxygenase, and further activates downstream signaling pathways such as PI3K/Akt. Thus, arachidonic acid plays an important role in the regulation of tumor cell proliferation and apoptosis. COX-2 is often over-expressed in tumor cells, which results in the accumulation of a large amount of PGE2 in tumor tissues (Łuczaj et al., 2015). PGE2 can inhibit tumor cell apoptosis, promote cell division, angiogenesis, tumor invasion and metastasis by binding to special receptors on the cell membrane. In addition, cPLA2 catalyzes membrane phospholipids to produce AA and lysophospholipids, which directly or indirectly participate in tumorigenesis and development (Clay et al., 1999; Sabino et al., 2002). This study found that the content of AA in the model group mice was increased, indicating that lung cancer caused severe inflammation in the body and may exist cancer cell division, angiogenesis, tumor invasion and metastasis. Andro can reduce AA levels by regulating arachidonic acid metabolism. Metabolite products such as lactic acid level is significantly increased in cancer patients, while glucose level is significantly decreased due to the continuous existence of the Warburg effect, which is the result of abnormal metabolization in tumor cells, that is, the strong Glucose metabolism. Cancer cell metabolism mainly involves the final conversion of glucose to lactate through enzyme-catalyzed anaerobic fermentation (Fan et al., 2009; Rocha et al., 2010; Rocha et al., 2011). Lactic acid is also the cyclic carbon source of tricarboxylic acid (TCA) for non-small cell lung cancer to maintain tumor metabolism in the body. Therefore, the elevated lactate levels found in the serum of patients with non-small cell lung cancer can be attributed to the large number of cell proliferations. Our research has found that the lactic acid content in the urine of lung cancer mice was increased, which showed that the tumor metabolism in cancer animals was accelerated. After Andro treatment, the abnormal lactic acid content was reduced mainly achieved by regulating pyruvate metabolism. In the human body, thymine participates in numerous enzymatic reactions, which thymine and deoxyribose 1-phosphate can be biosynthesized from thymidine through interaction with thymidine phosphorylase. In addition, it can be also converted to dihydrothymine (Jordan et al., 2010; Faubert et al., 2017).

Thymine is a potentially toxic compound that is associated with numerous diseases, such as thymidine treatment, periodontal detection depth, colorectal cancer and temporomandibular joint disease in human. It is also associated with innate metabolic disease of β-uropropionase deficiency (Xu et al., 2016). Pathway analysis results have shown that Andro can reduce abnormally elevated levels of thymine in urine by regulating pyrimidine metabolism. As a kind of porphyrin, coporphyrin III enters mitochondria, where it is oxidized and decarboxylated to form protoporphyrin IX. It is catalyzed by iron chelatase, which combines Fe^2+^ with protoporphyrin-IX to form heme. Drug toxicity can cause liver damage and hemoglobin synthesis dysfunction resulting to the increasing level of the synergistic porphyrin III in the urine, which in turn causes abnormal bilirubin metabolism and increases the level of DBIL (Bröer, 2008; Deja et al., 2014). The level of coproporphyrin III in the urine of lung cancer mice is elevated, which shows that lung cancer may cause liver damage and hemoglobin synthesis dysfunction. After Andro treatment, the abnormal content of coproporphyrin III was changed to the horizontal direction of the control group. Inositol, as a reactant of tumor cell energy metabolism and lipid metabolism, is significantly increased when the body's immune function is low and tumor cell proliferation. Tumor energy, carbohydrates, and lipid metabolism in patients are more active, and their immune function protecting against tumor cells proliferation is faster. Some studies have added two other characteristics of cancer, namely reprogramming energy metabolism and evading immune destruction. In tumors from animal model, various energy metabolism pathways such as such as inositol phosphate metabolism, oxidative phosphorylation and purine metabolism and citrate cycle have changed. Inositol phosphate metabolism is altered in cancers body, and then they regulate chromatin remodeling (Steger et al., 2003; Hanahan and Weinberg, 2011). Andro can reduce inositol phosphate content in the urine of model mice by phosphatidylinositol signaling system and Inositol phosphate metabolism indicating that Andro can inhibit the energy, carbohydrate and lipid metabolism of tumor cells. Branched-chain amino acids (BCAA) such as valine, isoleucine and leucine possess similar structures with different metabolic pathways. Valine deficiency is marked by impaired brain nerve function, and isoleucine deficiency is marked by muscle tremors. Studies have reported that glycine, valine and methionine in the serum of lung cancer patients are significantly lower than those in healthy controls, which are considered to be essential in the development of primary tumor types (Khunger et al., 2018). 3-Hydroxybutyric acid, also known as β-hydroxybutyric acid, is a typical partial degradation product of branched-chain amino acids released from muscles for liver and kidney gluconeogenesis (Hashim et al., 2019). 12,13-EpOME is a very hydrophobic long-chain fatty acid. During the occurrence and development of tumors, a large amount of energy and raw materials are needed to meet the needs of their own growth due to the rapid metabolism of tumor cells, which will lead to an increase in fatty acid oxidation products in the body and a decrease in fatty acid content. This study found that the level of 12,13-EpOME in urine was increased, indicating that there may be serious abnormalities in lipid metabolism in cancer bodies. Xanthine acid as a metabolite of tryptophan catabolism is the substrate of methyltransferase in the tryptophan metabolism pathway. Xanthine is a product of the purine degradation pathway and will be converted to uric acid under the action of xanthine oxidase. p-Cresol sulfate that causes nephrotoxicity and vascular toxicity by activating the renal renin-angiotensin-aldosterone system (RAAS), and leads to renal tubular cell stress response cells and renal fibrosis is a uremic toxin (Battelli et al., 2018; Battelli et al., 2019). Creatinine is a breakdown product of creatine phosphate in muscles. The loss of water molecules in creatine leads to the formation of creatinine. Creatinine is transferred to the kidneys through plasma, and then cleared from the body through glomerular filtration and partial renal tubular excretion (Evans et al., 2019). ALK rearrangements result from inversions or translocations on chromosome 2 that fuse variable regions of a partner gene with exon 20 of the ALK gene (Shaw et al., 2009; Gainor et al., 2013; Tsao et al., 2016).

As an integral part of systems biology, metabolomics has developed rapidly in recent years. At this stage, metabolomics research in lung cancer is mainly focused on metabolic pathways of blood, urine, tissue cells, and breathing gas, while sputum and pleural effusion are rarely reported in the literature, and further research is needed (Crutchfield et al., 2016; Dakappagari et al., 2017; Li et al., 2018). Since the existing analytical instruments, analytical techniques, and data processing methods are not perfect, and specimen preparation lacks uniform standards, metabolomics technology still needs further development. With the continuous insight into metabolomics research, HMDB improvement, the successful docking of various omics data and the verification of multiple biological models, panoramic information on the transcription, protein and metabolic levels of various tumors such as lung cancer could be obtained, and more molecular markers for early diagnosis, efficacy and prognosis evaluation will be discovered providing a theoretical basis for improving the clinical diagnosis and treatment of lung cancer.

## Conclusion

This study discovers biomarkers, metabolic profiles and pathways as potential targets for insight into the pharmacological action and effective mechanism of Andro against lung cancer by high-throughput metabolomics analysis combined with network pharmacology. Andro can regulate 18 of 25 biomarkers associated with the pathogenesis of lung cancer, such as alanine, L-glutamine, isoleucine and 3-hydroxybutyric acid. Andro embodies the characteristics of enhancing the immune system function, inhibiting inflammation reaction, tumor cell growth and metastasis as well as balancing visceral metabolism, which was involved in amino acid metabolism, arachidonic acid metabolism, porphyrin and chlorophyll metabolism, pyruvate metabolism, pyrimidine metabolism, phosphatidylinositol signaling system and inositol phosphate metabolism. Andro were shown to address multiple relevant targets and signaling pathways in the Lewis lung cancer model. Due to generating the majority of biological data. Further, it could expand the number of biological samples and perform clinical biological verification in the research process of lung cancer.

## Data Availability

The original contributions presented in the study are included in the article/Supplementary Material, further inquiries can be directed to the corresponding authors.
