# Supplementary material for: Andrographolide Against Lung Cancer-New Pharmacological Insights Based on High-Throughput Metabolomics Analysis Combined with Network Pharmacology
Source: Front Pharmacol. 2021 Apr 21;12:596652. doi: 10.3389/fphar.2021.596652 (PMC8097142; doi:10.3389/fphar.2021.596652)
Supplement: Supplementary file 1 [file datasheet1.docx]

**Table S1** The distinct metabolites identified from urine samples in lung cancer animals model.

| **No.** | **Chemical**  **name** | **HMDB ID** | **Ion mode** | **Formula** | **m/z** | **RT**  **(min)** | **VIP value** | **Trend in model** | **Andro regulation** |
| --- | --- | --- | --- | --- | --- | --- | --- | --- | --- |
| 1 | Valine | HMDB00883 | [M+H] + | C5H11NO2 | 118.0862 | 0.52 | 2.68 | ↓ | √ |
| 2 | Inositol phosphate | HMDB02985 | [M+H] + | C6H13O9P | 261.0380 | 0.87 | 3.98 | ↑ | √ |
| 3 | Alanine | HMDB00161 | [M+H] + | C3H7NO2 | 90.0553 | 1.08 | 2.71 | ↓ | √ |
| 4 | Thymine | HMDB00262 | [M+H] + | C5H6N2O2 | 127.0502 | 1.14 | 6.34 | ↑ | √ |
| 5 | Proline | HMDB00162 | [M-H] - | C5H9NO2 | 114.0551 | 1.27 | 3.86 | ↓ | √ |
| 6 | L-Glutamine | HMDB00641 | [M-H] - | C5H10N2O3 | 145.0613 | 2.21 | 2.71 | ↓ | √ |
| 7 | Pyridoxic acid | HMDB00017 | [M-H] - | C8H9NO4 | 182.0455 | 2.25 | 1.65 | ↓ | × |
| 8 | 3-hydroxybutyric acid | HMDB00357 | [M-H] - | C4H8O3 | 103.0402 | 2.68 | 3.28 | ↓ | √ |
| 9 | Arginine | HMDB00517 | [M+H] + | C6H14N4O2 | 175.1193 | 3.11 | 2.71 | ↓ | √ |
| 10 | Arachidonic acid | HMDB0001043 | [M-H] - | C20H32O2 | 349.2378 | 3.55 | 9.03 | ↑ | √ |
| 11 | Xanthurenic acid | HMDB00881 | [M-H] - | C10H7NO4 | 204.0278 | 3.76 | 2.32 | ↓ | √ |
| 12 | Glucose | HMDB00122 | [M+H] + | C6H12O6 | 181.0711 | 3.90 | 4.65 | ↓ | × |
| 13 | Isoleucine | HMDB00172 | [M+H] + | C6H13NO2 | 132.1005 | 4.18 | 1.75 | ↓ | √ |
| 14 | p-Cresol sulfate | HMDB11635 | [M-H] - | C7H8O4S | 187.0062 | 4.44 | 2.71 | ↓ | √ |
| 15 | Kynurenic acid | HMDB00715 | [M+H] + | C10H7NO3 | 190.1675 | 5.37 | 2.71 | ↓ | √ |
| 16 | Tyrosine | HMDB00158 | [M+H] + | C9H11NO3 | 182.0808 | 5.76 | 1.46 | ↓ | √ |
| 17 | Chenodeoxycholic acid | HMDB00518 | [M+H] + | C24H40O4 | 393.5720 | 6.12 | 8.91 | ↑ | × |
| 18 | Creatinine | HMDB00562 | [M+H] + | C4H7N3O | 114.0667 | 6.34 | 1.87 | ↑ | √ |
| 19 | Phenylpyruvic acid | HMDB00205 | [M+H] + | C9H8O3 | 165.0561 | 6.59 | 4.28 | ↑ | × |
| 20 | Coproporphyrin III | HMDB01261 | [M-H] - | C36H44N4O8 | 659.3147 | 7.05 | 3.05 | ↑ | √ |
| 21 | 12,13-EpOME | HMDB04702 | [M+H] + | C18H32O3 | 297.2393 | 7.53 | 7.98 | ↑ | √ |
| 22 | Glycyl-Threonine | HMDB28851 | [M-H] - | C6H12N2O4 | 175.0744 | 7.80 | 2.05 | ↓ | × |
| 23 | 9(S)-HPODE | HMDB06940 | [M-H] - | C18H32O4 | 311.2232 | 8.13 | 8.51 | ↑ | × |
| 24 | 3-Oxohexadecanoic acid | HMDB10733 | [M-H] - | C16H30O3 | 269.2092 | 8.32 | 1.91 | ↑ | × |
| 25 | Lactic acid | HMDB00190 | [M-H] - | C3H6O3 | 89.0234 | 9.14 | 2.34 | ↑ | √ |


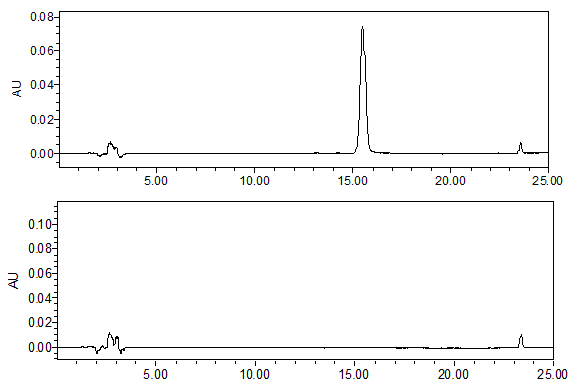


**Figure S1**  HPLC chromatographic fingerprint of Andro. The chromatographic conditions were ACQUITY UPLC BEH C18 (100 mm×2.1 mm, 1.7μm) chromatographic column, using acetonitrile-water as mobile phase gradient elution, flow rate of 0.3 mL•min-1, column temperature of 30 ℃, detection wavelength of 225 nm (andrographolide)


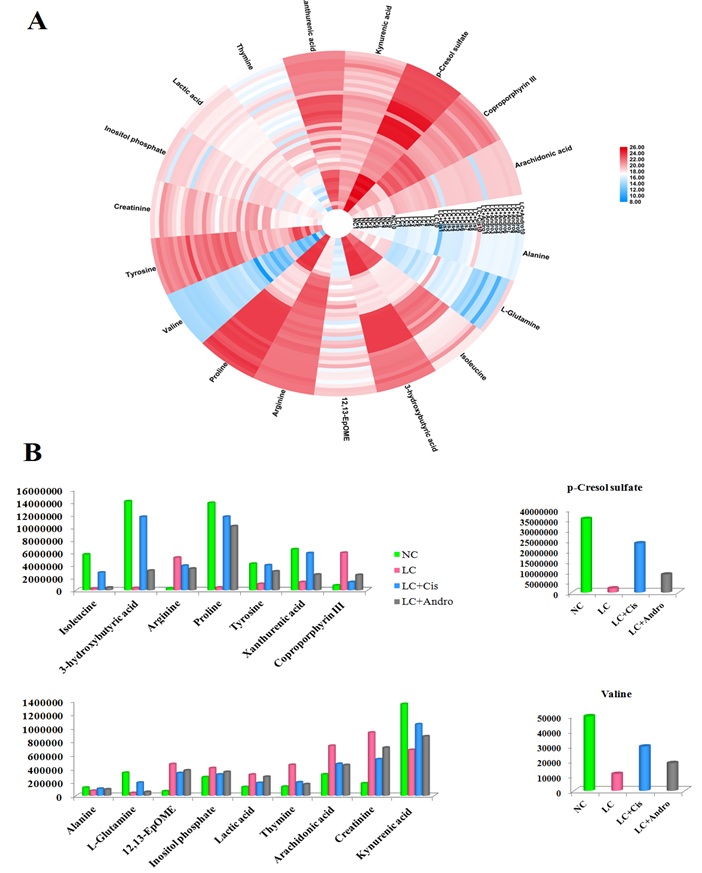


Figure S2 A heatmap to describe the changed trend in NC, LC, LC+Cis and LC+Andro group (A). The red areas indicate an increasing trend, and the blue areas indicate a decreasing trend; Average peak area changes of 18 potential metabolites in NC, LC, LC+Cis and LC+Andro group (B).
